# Water‐Wave Pancharatnam‐Berry Phase Induced by 4D Spin‐Orbit State Evolution

**DOI:** 10.1002/advs.202515337

**Published:** 2025-10-13

**Authors:** Wanyue Xiao, Shiqi Jia, Tong Fu, Shubo Wang

**Affiliations:** ^1^ Department of Physics City University of Hong Kong Tat Chee Avenue, Kowloon Hong Kong China

**Keywords:** angular momentum, geometric phase, Pancharatnam‐Berry phase, water wave

## Abstract

Geometric phase is a far‐reaching concept in quantum and classical physics, revealing deep connections between topology and wave dynamics in diverse systems. The Pancharatnam‐Berry (PB) geometric phase, which arises from 2D optical polarization evolution, has revolutionized light manipulation by enabling optical metasurfaces. The PB phase is commonly regarded as a unique property of light waves. Generalizing this concept to other classical waves can uncover new geometric phase properties and enable novel wave control mechanisms. Here, the first observation of the PB phase in surface water (gravity) waves is reported, achieved through the interaction with symmetry‐engineered scatterers. In contrast to the optical PB phase, the water‐wave PB phase is an intrinsic higher‐order geometric phase induced by 4D evolution of spin‐orbit states. It exhibits remarkable properties, including unbounded phase values, spin‐momentum‐locked unidirectional channels, and a Poincaré hypersphere representation. PB metasurfaces are further realized for versatile manipulation of water wavefronts, including steering and focusing. This work reveals the universality of the PB phase across different wave systems and generalizes it to higher‐dimensional state evolutions. The results open new avenues for exploring the geometric and topological properties of water waves, with potential applications in coastal protection and wave energy harvesting.

## Graphical abstract

The first observation of the Pancharatnam‐Berry (PB) phase in water waves is reported, revealing its extraordinary properties including unbounded phase values, spin‐momentum‐locked unidirectional channels, and a Poincaré hypersphere representation. These findings demonstrate the universality of the PB phase across different wave systems and in higher‐dimensional state evolutions, with promising applications in coastal protection and wave energy harvesting.

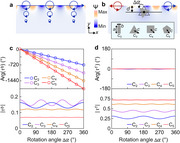

## 1 Introduction

Geometric phases arise from state evolutions in parameter space and are ubiquitous in quantum and classical systems, underpinning fundamental physical phenomena from the Aharonov‐Bohm effect^[^ ^1^, ^2^ ^]^ to topological states.^[^ ^3^, ^4^, ^5^ ^]^ Discovered by S. Pancharatnam^[^ ^6^ ^]^ and generalized by M. V. Berry,^[^ ^7^ ^]^ the Pancharatnam‐Berry (PB) geometric phase can enable subwavelength control of optical wavefront and has revolutionized light manipulation,^[^ ^8^, ^9^, ^10^ ^]^ ushering in the era of flat optics with metasurface‐empowered ultra‐thin optical devices.^[^ ^11^, ^12^, ^13^, ^14^, ^15^ ^]^ The integration of artificial intelligence with the PB phase has further expanded the capabilities of metasurfaces.^[^ ^16^, ^17^ ^]^ Unlike the momentum‐space geometric phases,^[^ ^3^, ^4^, ^5^ ^]^ the PB phase emerges due to optical polarization evolution in a 2D space represented by the Poincaré sphere. Generalizing the PB phase to other classical waves can uncover new properties of the geometric phase and open new avenues for wave manipulation.

Water waves, easily accessible yet dynamically richer than light waves, provide an ideal platform to explore the universality of the PB phase across different wave systems. Unlike the transverse electromagnetic nature of light, water waves are surface waves with a hybrid transverse‐longitudinal characteristic. Beyond their natural abundance and renewable energy potential, water waves exhibit a variety of intriguing phenomena, such as cloaking,^[^ ^18^, ^19^ ^]^ superlensing,^[^ ^20^ ^]^ holography,^[^ ^21^ ^]^ surface polaritons,^[^ ^22^ ^]^ and topological quasiparticles.^[^ ^23^, ^24^ ^]^ Owing to their easy accessibility and straightforward observation, water waves are also suitable for investigating fundamental wave phenomena that are universal across classical systems, including Cherenkov radiation,^[^ ^25^ ^]^ time‐reversal dynamics,^[^ ^21^, ^26^ ^]^ and spatiotemporal vortices.^[^ ^27^ ^]^ Despite significant progress in the control and manipulation of water waves,^[^ ^28^, ^29^, ^30^, ^31^, ^32^, ^33^, ^34^, ^35^, ^36^, ^37^, ^38^ ^]^ the PB phase has never been observed in water‐wave systems. The fundamental differences between water waves and light render the generalization of the PB phase to water waves highly nontrivial.

Here, we report the first experimental observation of the PB phase in water waves. By leveraging the interaction with subwavelength anisotropic scatterers, we uncover how the new PB phase arises from the coupled evolution of spin and orbital angular momentum (OAM) of water waves in a 4D state space, which can be represented on the Poincaré hypersphere composed of three nested higher‐order Poincaré spheres (HOPSs), thus generalizing the PB phase to *higher‐dimensional* state evolutions for the first time. In contrast to the conventional optical PB phase, the water‐wave PB phase is an intrinsic higher‐order geometric phase and exhibits remarkable properties, such as unbounded phase values and unidirectionality due to the spin‐momentum locking. Harnessing this phase, we realize PB metasurfaces that enable nearly arbitrary water wavefront manipulation, including steering and focusing.

## 2 Results

### 2.1 Water Wave Scattering

**Figure 1:**
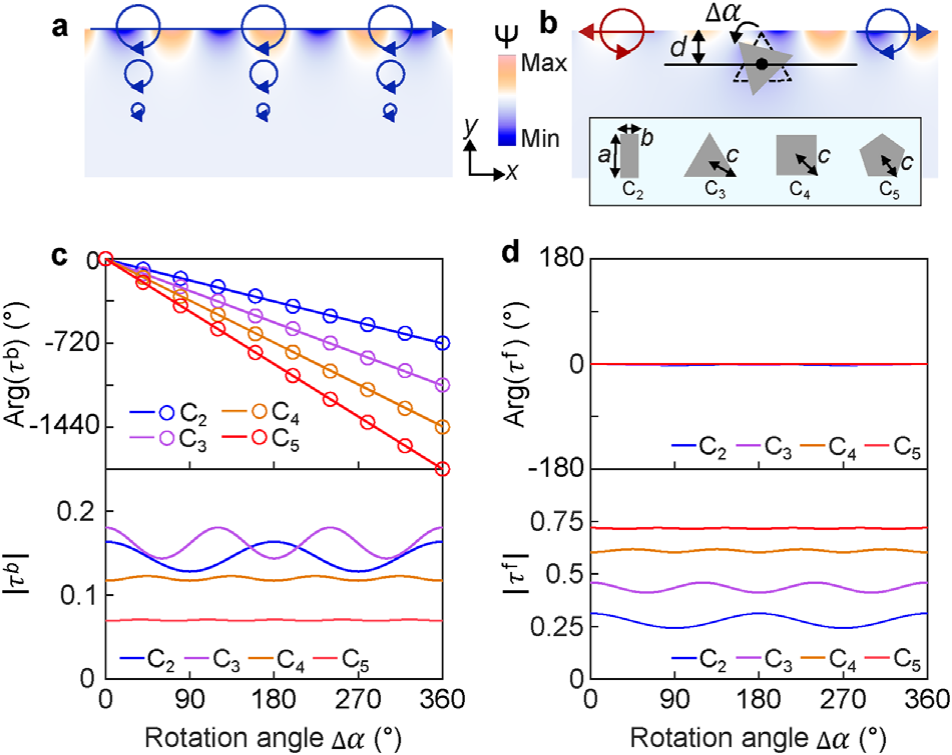
PB phase of water waves. a) Velocity potential and polarization ellipses for the water wave propagating in +x direction. b) Scattering of the incident water wave by scatterers with different rotational symmetries. c) Phase and amplitude of the backward scattering coefficient. The solid lines denote the simulation results. The circles denote the analytical results. d) Phase and amplitude of the forward scattering coefficient.

We consider harmonic water waves propagating at the surface (y=0) of linear, inviscid, and irrotational deep water. The velocity field $\left|v\right\rangle =\left(\hat{x}+i\sigma \hat{y}\right)e^{-i\sigma \mathrm{kx}+\mathrm{ky}}$ (k is the popagation constant) is the eigenmode of the spin‐1 operator ${\hat{S}}_{z}$ with eigenvalue σ=±1, exhibiting an intrinsic property of spin‐momentum locking, i.e., |v⟩ with opposite spin propagate in opposite directions.^[^ ^35^, ^39^ ^]^ **Figure** **1**a shows the velocity potential Ψ(|v⟩=∇Ψ) for the water wave propagating in +x direction and carrying transverse spin in −z direction, where the circles denote the polarization ellipses of the vector velocity field, corresponding to the circular motion of water particles. When it impinges on a subwavelength scatterer immersed at depth d, both forward and backward scattering waves are generated, as shown in Figure 1b. The scatterer has rotational symmetry *C*_*m*_ and is infinitely extended in z direction, with orientation characterized by the rotation angle Δα. Figure 1c and d show the numerically simulated forward ($\tau^{f}$) and backward ($\tau^{b}$) scattering coefficients, respectively, as a function of Δα for the four scatterers in the inset of Figure 1b. The amplitudes |$\tau^{f}$| and |$\tau^{b}$| generally vary with Δα. Interestingly, the phase of $\tau^{b}$ exhibits a linear dependence on the rotation angle, i.e., $\mathrm{Arg}(\tau^{b})=-m\Delta \alpha$, while the phase of $\tau^{f}$ is not affected by the rotation angle.

Partial wave analysis can explain the phase properties of the scattering coefficients. Using the local polar coordinates (r,α), the velocity field around the scatterers can be expanded as^[^ ^40^ ^]^:

(1) $$\left|v\right\rangle =\sum_{n=0}^{+\infty }\left|v_{n}\right\rangle =\sum_{n=0}^{+\infty }I\left(\hat{x}+i\sigma \hat{y}\right)\frac{{\left(-i\sigma kr\right)}^{n}e^{i\sigma n\alpha }}{n!},$$

where the integer n is the expansion order and I characterizes the amplitude of the partial waves. The partial waves $|v_{n}\rangle$ are eigenmodes of the spin‐1 operator ${\hat{S}}_{z}$ and OAM operator ${\hat{L}}_{z}$ with eigenvalues σ and l=σn, respectively. They carry total angular momentum $j_{n}=\sigma +\sigma n$ in z direction. Specifically, the partial waves propagating in +x(−x) direction have σ=−1 (σ=+1) and thus $j_{n}=-1-n<0$ ($j_{n}=1+n>0$). Using Equation (1) and angular momentum selection rule,^[^ ^41^, ^42^ ^]^ the forward and backward scattering coefficients can be expressed as

(2) $$\tau^{f}=\sum_{j_{n^{\prime }}^{f}-j_{n}^{i}=0}\langle v_{n^{\prime }}^{f}|\hat{T}\left|v_{n}^{i}\right\rangle ,$$

(3) $$\tau^{b}=\sum_{j_{n^{\prime }}^{b}-j_{n}^{i}=m}\left\langle v_{n^{\prime }}^{b}\right|\hat{T}|v_{n}^{i}\rangle e^{-im\Delta \alpha },$$

where $|v_{n}^{i}\rangle ,|v_{n^{\prime }}^{f}\rangle$, and $|v_{n^{\prime }}^{b}\rangle$ are the partial waves of the incident field, forward scattering field, and backward scattering field, respectively, which carry the total angular momentum $j_{n}^{i},j_{n^{\prime }}^{f}$, and $j_{n^{\prime }}^{b}$. $\hat{T}$ characterizes the interaction of the scatterer with the incident field in the scatterer's local frame. Equation (2) indicates that $\mathrm{Arg}(\tau^{f})$ is independent of the rotation angle Δα, consistent with the simulation results in Figure 1d. In contrast, Equation (3) indicates that $\tau^{b}$ carries an additional phase −mΔα corresponding to the circles in Figure 1c, agreeing with the simulation results there. This phase is a higher‐order PB phase^[^ ^43^, ^44^ ^]^ induced by the evolution of total angular momentum: $\varphi_{\mathrm{PB}}=\left(j_{n}^{i}-j_{n^{\prime }}^{b}\right)\Delta \alpha$, which exists for any scatterers with discrete rotational symmetries.

### 2.2 Angular Momentum Evolution

**Figure 2:**
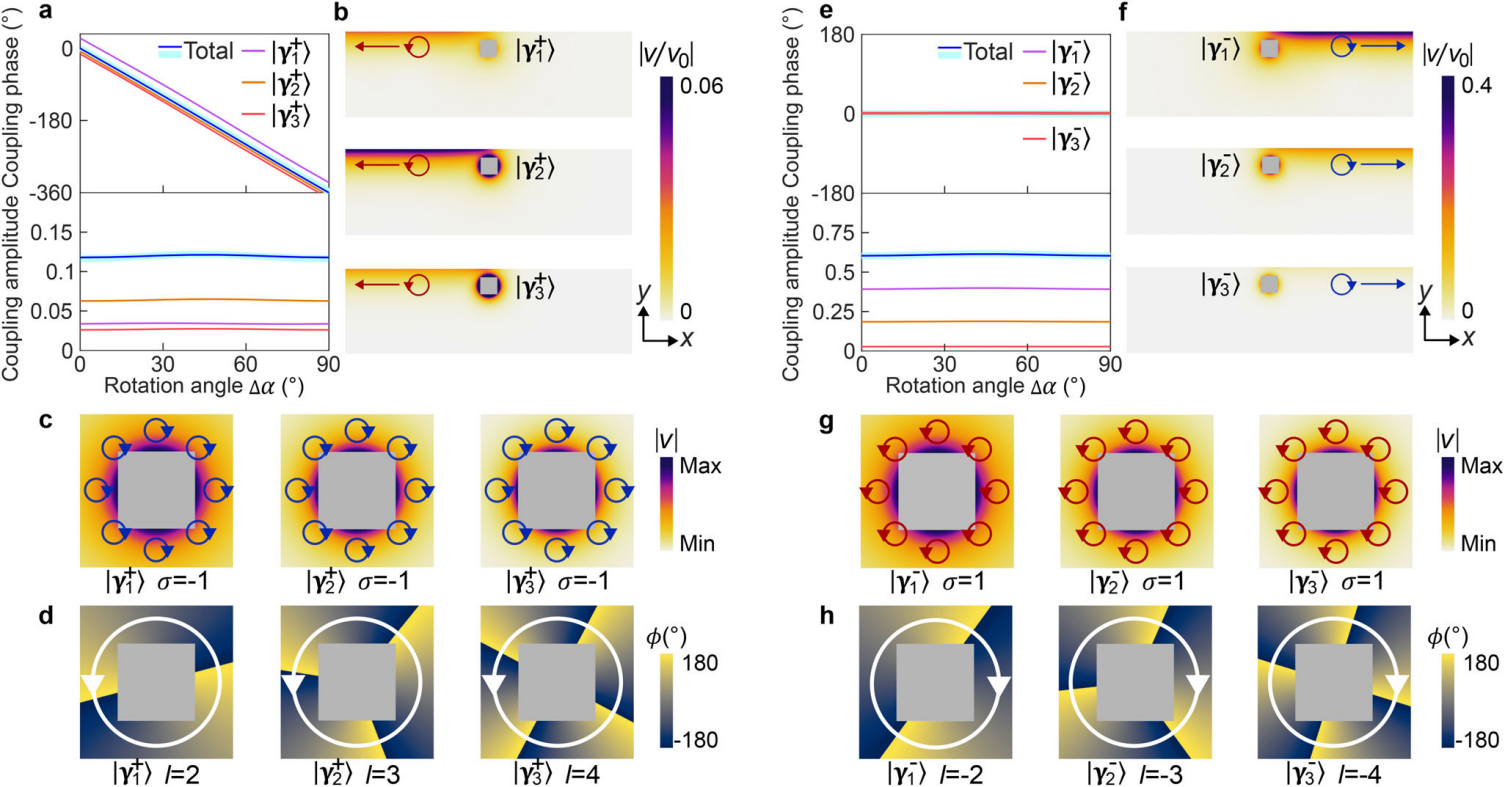
Multipoles excited in the scatterer. a) Coupling phases and amplitudes of the multipoles $|\gamma_{1}^{+}\rangle ,|\gamma_{2}^{+}\rangle$ and $|\gamma_{3}^{+}\rangle$. The light blue line denotes the total coupling from the three multipoles. The dark blue line denotes the backward scattering coefficient. b) Water waves coupled from the multipoles. c) Amplitudes and polarizations of the eigen‐velocity fields of the multipoles. The blue circles denote the polarization ellipses. d) Phases of the eigen‐velocity fields. e) Coupling phases and amplitudes of the multipoles $|\gamma_{1}^{-}\rangle ,|\gamma_{2}^{-}\rangle$ and $|\gamma_{3}^{-}\rangle$. f) Water waves coupled from the multipoles. g) Amplitudes and polarizations of the eigen‐velocity fields of the multipoles. The red circles denote the polarization ellipses. h) Phases of the eigen‐velocity fields.

The scatterer interacts with the water wave and induces the angular momentum evolution. To understand this, we apply multipole expansion to the field of the scatterer and analyze the angular momenta of the excited multipoles. The velocity field |γ⟩ of the scatterer can be expanded into two types of multipoles with amplitudes $a_{n}$ and $b_{n}$ (Note S3 and S4, Supporting Information):

(4) $$\left|\gamma \right\rangle =\sum_{n=1}^{+\infty }a_{n}\left(\hat{x}-i\hat{y}\right)\frac{e^{i(n+1)\alpha }}{k^{n}r^{n+1}}+b_{n}\left(\hat{x}+i\hat{y}\right)\frac{e^{-i(n+1)\alpha }}{k^{n}r^{n+1}},$$

where the first type multipoles, defined as $|\gamma_{n}^{+}\rangle$, carries positive total angular momentum $j_{n}^{+}=n$; the second type multipoles, defined as $|\gamma_{n}^{-}\rangle$, carries negative total angular momentum $j_{n}^{-}=-n$. As an example, we analyze the multipoles of the *C*_4_ scatterer and reveal their couplings with the water wave. By numerically evaluating $a_{n}$, we find that three dominant multipoles $|\gamma_{1}^{+}\rangle ,|\gamma_{2}^{+}\rangle$ and $|\gamma_{3}^{+}\rangle$ are excited in the scatterer. They unidirectionally couple to the water wave propagating in −x direction, as shown in **Figure** **2**b. The numerically obtained coupling phase and amplitude for each multipole are shown in Figure 2a. The total coupling of the three multipoles (light blue line) agrees with the backward scattering coefficient (dark blue line). Figure 2c shows the amplitudes and polarizations of the eigen‐velocity fields of the three multipoles. The fields are right‐handed circularly polarized with spin σ=−1. Figure 2d shows the phases of the fields, revealing different OAM of the multipoles: l=2 for $|\gamma_{1}^{+}\rangle$, l=3 for $|\gamma_{2}^{+}\rangle$, and l=4 for $|\gamma_{3}^{+}\rangle$.

By numerically evaluating $b_{n}$, we find that three dominant multipoles $|\gamma_{1}^{-}\rangle ,|\gamma_{2}^{-}\rangle$ and $|\gamma_{3}^{-}\rangle$ are also excited in the scatterer. They unidirectionally couple to the wave propagating in +x direction, as shown in Figure 2f. Figure 2e shows the numerically obtained coupling phase and amplitude for each multipole. The total coupling of the three multipoles (light blue line) agrees well with the forward scattering coefficient (dark blue line). Figure 2g,h display the polarizations and phases of the eigen‐velocity fields of the three multipoles, respectively. The fields are left‐handed circularly polarized with spin σ=1. They carry negative OAM: l=−2 for $|\gamma_{1}^{-}\rangle$, l=−3 for $|\gamma_{2}^{-}\rangle$, and l=−4 for $|\gamma_{3}^{-}\rangle$.

The excitation of the multipoles and their couplings with the water waves are governed by the selection rule and angular momentum conservation, respectively. The excited multipoles carry the total angular momentum $j_{n^{\prime \prime }}^{\pm }=j_{n}^{i}+mN\left(N\in Z\right)$ and couple to the scattering waves with $j_{n^{\prime }}^{b,f}=j_{n^{\prime \prime }}^{\pm }$. For the considered *C*_4_ scatterer with *m* = 4, we have $j_{n^{\prime \prime }}^{\pm }=j_{n^{\prime }}^{b,f}=j_{n}^{i}+4N$, which can be applied to determine the excited multipoles and scattering partial waves. For example, the incident partial wave with $j_{0}^{i}=-1$ excites the multiples $|\gamma_{1}^{-}\rangle$ with $j_{1}^{-}=-1$ and $|\gamma_{3}^{+}\rangle$ with $j_{3}^{+}=3$ under *N* = 0 and *N* = 1, respectively, which further couple to forward‐scattering partial wave with $j_{0}^{f}=-1$ and backward‐scattering partial wave with $j_{2}^{b}=3$. This process involves the evolutions of both spin σ and OAM l, as shown in the first row of **Table** **1**, where we label the multipoles and partial waves as |l,σ⟩. The same analysis can be applied to other multipoles in Figure 2 to uncover the angular momentum evolutions, which are also included in Table 1.

**Table 1:** Angular momentum evolutions for *C*_4_ scatterer.

Incident	Scatterer	Backward	Forward
		scattering	scattering
|0, −1〉	*b*_1_| − 2, 1〉 + *a*_3_|4, −1〉	|2, 1〉	|0, −1〉
| − 1, −1〉	*b*_2_| − 3, 1〉 + *a*_2_|3, −1〉	|1, 1〉	| − 1, −1〉
| − 2, −1〉	*b*_3_| − 4, 1〉 + *a*_1_|2, −1〉	|0, 1〉	| − 2, −1〉

### 2.3 Poincaré Hypersphere Representation

The polarization state of vector fields can be represented on the Poincaré sphere.^[^ ^45^, ^46^ ^]^ For fields carrying both spin and OAM, the complete state space (corresponding to a product space of the spin and OAM eigenstates) generally spans infinitive levels. When restricted to two levels, the states can be represented on a HOPS.^[^ ^43^, ^47^ ^]^ More generally, the states of a *N*‐level system can be represented on a Poincaré hypersphere comprising *N* − 1 nested HOPSs.^[^ ^48^, ^49^ ^]^ The Poincaré hypersphere representation can illustrate the geometric nature of the water‐wave PB phase.

**Figure 3:**
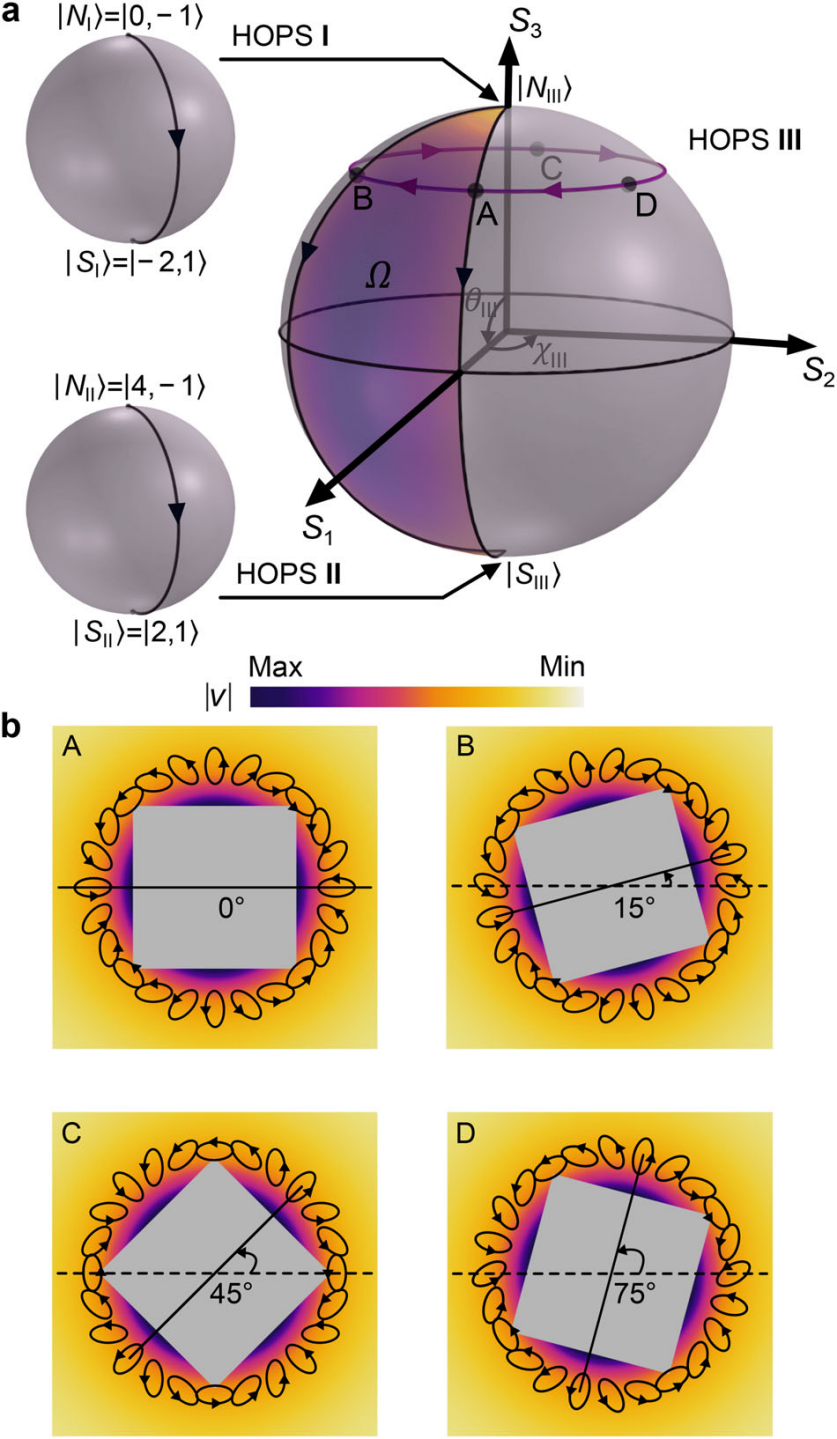
Poincaré hypersphere representation. a) Poincaré hypersphere composed of three coupled HOPSs. Each HOPS has a north pole and a south pole. The north (south) pole of HOPS III corresponds to an arbitrary state on HOPS I (II). The state evolutions in the water wave scattering trace out paths (black lines with arrows) on the three HOPSs. A variation of the scatterer's rotation angle changes its state from point A to point B. The PB phase equals half of the solid angle Ω subtended by the purple area on HOPS III. b) The state of the scatterer at the points A, B, C, D on HOPS III, corresponding to Δα = 0°, 15°, 45°, and 75°, respectively. The black ellipses show the spatial polarization distribution of the velocity field.

We consider the state evolution in the first row of Table 1 as an example. The principles also apply to other cases. The system has a 4D Hilbert space spanned by |0, −1〉, | − 2, 1〉, |4, −1〉, and |2, 1〉. In this case, the Poincaré hypersphere comprises three nested HOPSs, as shown in **Figure** **3**a. Each HOPS features a north pole state |*N*_*i*_〉 and a south pole state |*S*_*i*_〉, and any other point on the sphere denotes a superposition state $|\psi \left(\theta_{i},\chi_{i}\right)\rangle =\cos\left(\theta_{i}/2\right)|N_{i}\rangle +\sin\left(\theta_{i}/2\right)e^{i\chi_{i}}\left|S_{i}\right\rangle$ characterized by the polar angle *θ*_*i*_ and azimuthal angle *χ*_*i*_. In our settings, HOPS I has two pole states |*N*_I_〉 = |0, −1〉 and |*S*_I_〉 = | − 2, 1〉; HOPS II has two pole states |*N*_II_〉 = |4, −1〉 and |*S*_II_〉 = |2, 1〉. HOPS I and HOPS II are embedded into the north pole and south pole of HOPS III, respectively, i.e., |*N*_III_〉 (|*S*_III_〉) represents an arbitrary state on HOPS I (II). Thus, HOPS III characterizes the coupling of HOPS I and HOPS II with *θ*_III_ and *χ*_III_, which enables the representation of arbitrary superposition state in the entire 4D space.

The state evolution associated with backward scattering, i.e., |0, −1〉 → $b_{1}$| − 2, 1〉 + $a_{3}$|4, −1〉 → |2, 1〉, can be represented on the Poincaré hypersphere as follows. The incident state |0, −1〉 corresponds to |*N*_III_〉 = |*N*_I_〉. The excitation of the scatterer induces the evolution |0, −1〉 → $b_{1}$| − 2, 1〉 + $a_{3}$|4, −1〉, which traces out the paths *N*_I_ → *S*_I_ on HOPS I and *N*_III_ → A on HOPS III. Here, the point A locates on HOPS III with |*N*_III_〉 = |*S*_I_〉 and |*S*_III_〉 = |*N*_II_〉, and it represents the state $b_{1}$| − 2, 1〉 + $a_{3}$|4, −1〉 of the scatterer at rotation angle Δα=0. The coupling from the scatterer to the backward scattering wave further induces the evolution $b_{1}$| − 2, 1〉 + $a_{3}$|4, −1〉 → |2, 1〉, which traces out the paths *N*_II_ → *S*_II_ on HOPS II and A → *S*_III_ on HOPS III, where |*S*_III_〉 = |*S*_II_〉 represents the backward scattering state. Therefore, the full evolution process traces out a geodesic path on each HOPS from north pole to south pole, as shown in Figure 3a. When the scatterer rotates by angle Δα, its state changes from point A to point B. The resulting PB phase equals half of the solid angle Ω subtended by the area enclosed by the loop *N*_III_ → A → *S*_III_ → B → *N*_III_ on HOPS III. Notably, the state evolutions on HOPS I and HOPS II do not form closed loops and, thus, do not contribute to the PB phase. In addition, the evolution associated with the forward scattering, i.e., |0, −1〉 → $b_{1}$| − 2, 1〉 + $a_{3}$|4, −1〉 → |0, −1〉, traces out the path *N*_III_ → A → *N*_III_ → B → *N*_III_ on HOPS III, leading to Ω=0 and thus zero PB phase.

To intuitively understand the dependence of the PB phase on the rotation angle Δα, we show in Figure 3b the state of the scatterer at Δα = 0°, 15°, 45°, and 75°, corresponding to the points A, B, C, and D on HOPS III, respectively. The state has the same *C*_4_ symmetry and rotates with the scatterer. It returns to the original configuration after the scatterer rotates by Δα = 90°, i.e., A → B → C → D → A. Correspondingly, the evolution path sweeps a solid angle Ω=−720° on HOPS III. Thus, the PB phase is $\varphi_{\mathrm{PB}}=\Omega /2=-4\Delta \alpha$. The above interpretation reveals how the topology and geometry of the Poincaré hypersphere are imprinted on the phase of the water wave evolving in the 4D Hilbert space.

### 2.4 Wavefront Manipulation and Experiment

**Figure 4:**
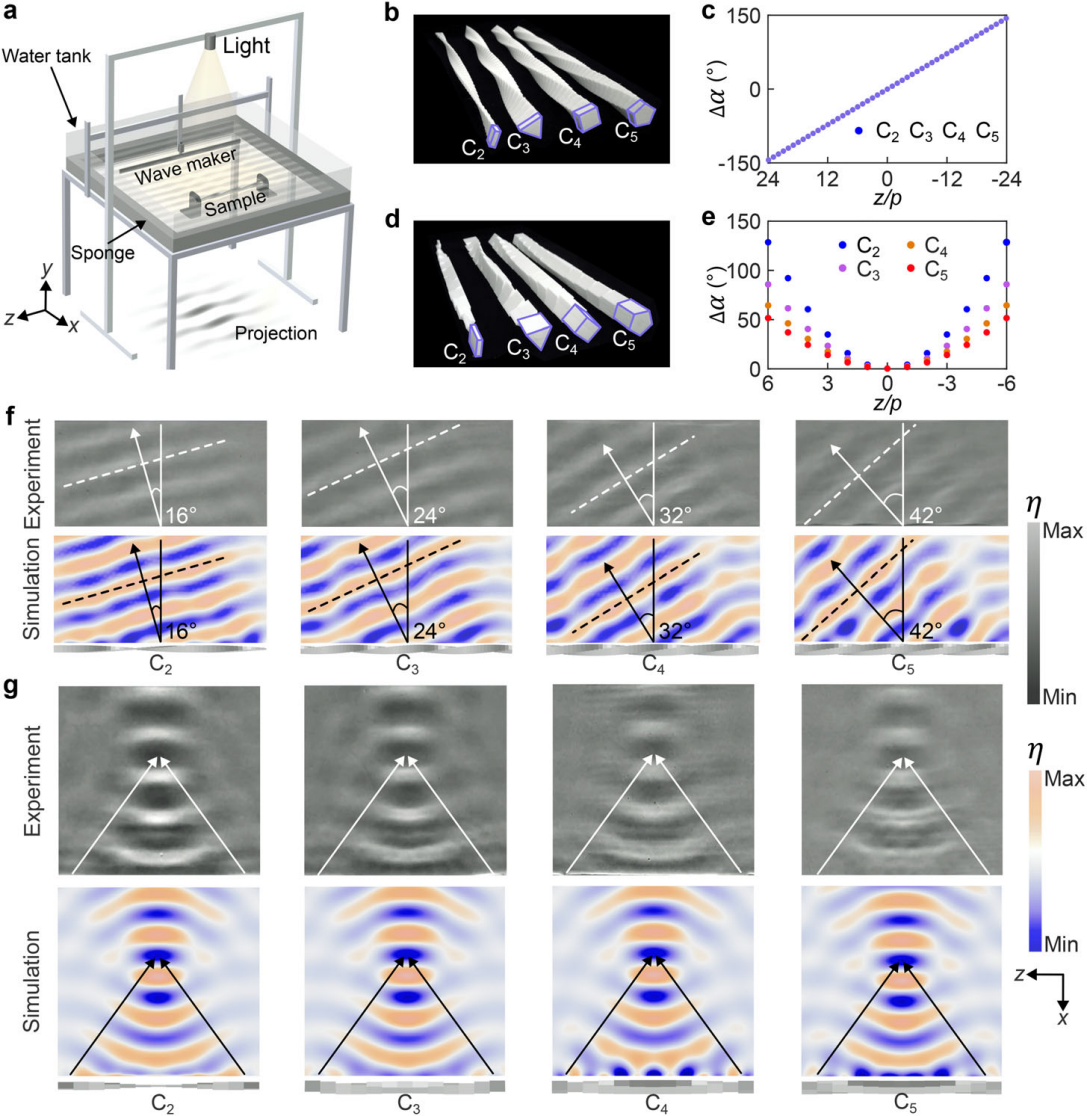
Wavefront manipulation by PB metasurfaces. a) Experimental setup. Photographs of the fabricated metasurface samples for b) deflection and d) focusing of water waves. The meta‐atoms (purple framed) have different rotational symmetries *C*_2_, *C*_3_, *C*_4_, and *C*_5_. Their rotation angles are shown in c,e), respectively, as a function of z coordinate. f) Experimentally measured (upper panels) and numerically simulated (lower panels) surface elevation of the water waves deflected by the metasurfaces in b). g) Experimentally measured (upper panels) and numerically simulated (lower panels) surface elevation of the water waves focused by the metalenses in d).

The PB phase can enable nearly arbitrary manipulation of water wavefront. We construct PB metasurfaces with the finite‐length scatterers and experimentally demonstrate anomalous deflection and focusing of water waves by using the setup in **Figure** **4**a. The water wave is generated by a wave maker and propagates toward the metasurface. A light source above the tank projects the wave pattern onto a plane beneath, which can be directly observed and video‐recorded for analysis.

To achieve anomalous deflection, we design four PB metasurfaces using the meta‐atoms with *C*_2_, *C*_3_, *C*_4_, and *C*_5_ symmetries, respectively, as shown in Figure 4b. All four metasurfaces have the same rotation angle profile Δα(z), as shown in Figure 4c. They induce the *m*‐dependent PB phase $\varphi_{\mathrm{PB}}\left(z\right)=-m\Delta \alpha \left(z\right)$ and phase gradient $k_{g}=\varphi_{\mathrm{PB}}/z$ and can deflect the incident water wave with angle $\beta =\sin^{-1}\left(k_{g}/k\right)$ according to the generalized Snell's law.^[^ ^50^ ^]^ Figure 4f shows the experimental and simulation results, where the incident water wave propagates downward (along +x direction). The upper panels show the measured surface elevation η=ReiωΨ/g of the backward water wave, which agree well with the simulation results in the lower panels. The predicted deflection angles are β = 16°, 24°, 32°, and 42° for the metasurfaces *C*_2_, *C*_3_, *C*_4_, and *C*_5_, respectively, which are consistent with the simulated and experimentally measured wavefronts. The water wave deflection induces a lateral force (along −z direction), in addition to the forward propelling force, on the metasurfaces. This mechanism can be employed to design watercrafts powered by water waves, with the travelling direction controlled with the PB geometric phase.

To achieve focusing of water waves, we design four PB metalenses with the same PB phase profile $\varphi_{\mathrm{PB}}\left(z\right)=-k\left(\sqrt{z^{2}+f^{2}}-f\right)(f$ is the focal length), as shown in Figure 4d. These metalenses have different rotation angle profiles Δα(z), as shown in Figure 4e, where Δα is smaller for the meta‐atoms with a higher rotational symmetry. Figure 4g shows the experimental and simulation results for the surface elevation of the backward water wave, where the incident wave propagates along +x direction. We observe that all the metalenses can focus the water wave at the same position with a focal length f=3.2λ, despite of their different rotation angle profiles Δα(z). The analytically predicted focal lengths based on the expression of $\varphi_{\mathrm{PB}}\left(z\right)$ are indicated by the arrows, which agree with the experimental and simulation results.

## 3 Conclusion

**Figure 5:**
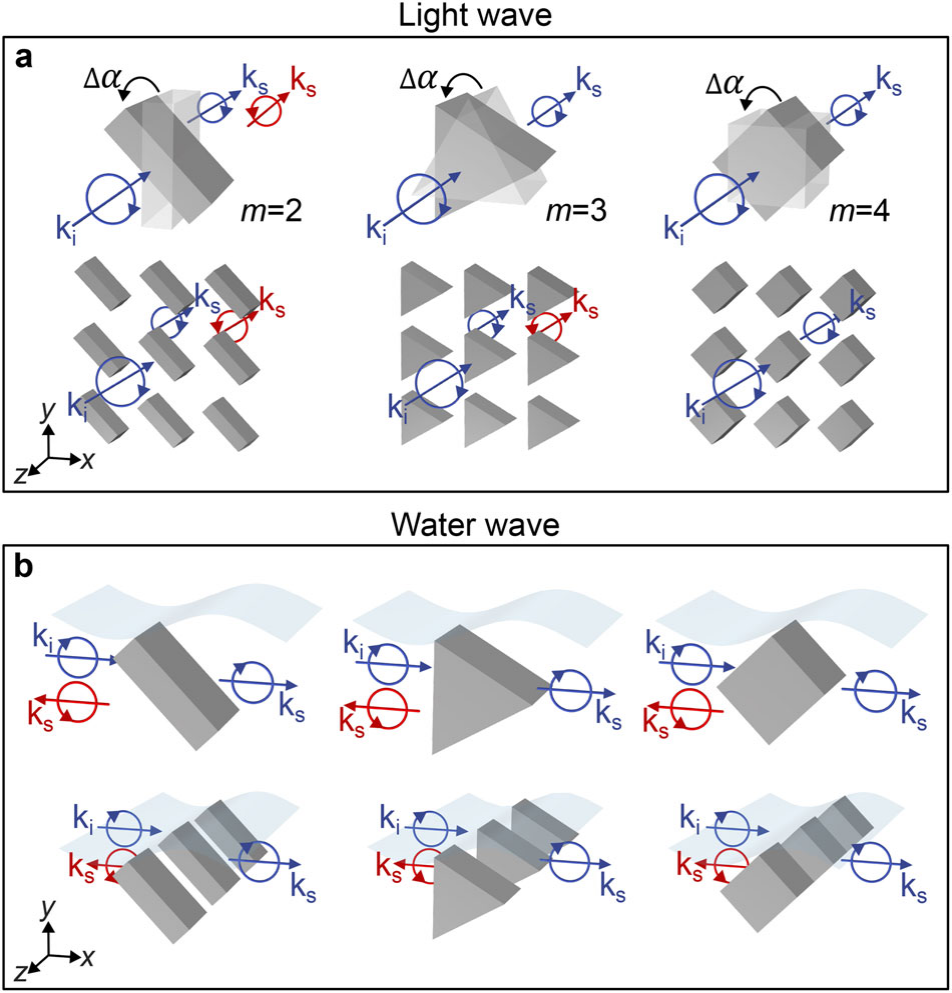
Comparison between optical PB phase and water‐wave PB phase. a) Circularly polarized light wave passing through a single scatterer (upper panel) and periodic scatterers (lower panel) with different rotational symmetries. For a single scatterer: $\varphi_{\mathrm{PB}}=-2\Delta \alpha$, if *m* ⩽ 2; $\varphi_{\mathrm{PB}}=0$, otherwise. For periodic scatterers: $\varphi_{\mathrm{PB}}=-m\Delta \alpha$, if *m* is even; $\varphi_{\mathrm{PB}}=-2m\Delta \alpha$, if *m* is odd; $\varphi_{\mathrm{PB}}=0$, if *m* = 4. b) Water wave scattered by a single scatterer (upper panel) and periodic scatterers (lower panel) with different rotational symmetries. In both cases, $\varphi_{\mathrm{PB}}=-m\Delta \alpha$ is observed in the backward‐scattering spin channel.

The water‐wave PB phase differs significantly from the conventional optical PB phase. For subwavelength scatterers under the incidence of a circularly polarized plane wave, as shown in **Figure** **5**a (upper panels), the optical PB phase emerges due to the flipping of the longitudinal spin, which equals twice the rotation angle of the scatterers and is decided by the differential spin of the incident and scattering lights. The phase disappears for the scatterers with *m* ⩾ 3 rotational symmetries, which are effectively isotropic for the incident plane wave.^[^ ^51^, ^52^ ^]^ Such scatterers can still induce an optical PB phase if arranged into a lattice such as the square lattices in Figure 5a (lower panels), which generally does not exhibit a simple dependence on the rotation angle of the scatterers due to the lattice‐coupling‐induced effective anisotropy.^[^ ^53^ ^]^ In contrast, the water‐wave PB phase emerges due to the flipping of the transverse spin, which can be induced by individual scatterers of arbitrary rotational symmetry, as illustrated in Figure 5b (upper panels). The phase exhibits remarkable unidirectionality due to the spin‐momentum locking. Importantly, the same PB phase also emerges in the periodic systems in Figure 5b (lower panels), because the rotation axis of the scatterers is parallel to the lattice. In addition, water waves are inherently inhomogeneous due to the water‐air interface. This inhomogeneity enables water waves to carry a higher‐order PB phase with Poincaré hypersphere representation, giving rise to rich physics associated with state evolutions in a higher‐dimensional space.

In summary, we demonstrate a new kind of PB phase in water waves through interaction with anisotropic scatterers. Unlike the conventional optical PB phase, the water‐wave PB phase exhibits intrinsic higher‐order characteristics arising from the synergy between OAM and spin in a higher‐dimensional space, with their evolutions represented on the Poincaré hypersphere. We apply this phase to realize PB metasurfaces and metalenses for nearly arbitrary manipulation of the water wavefronts, with potential applications in coastal protection and wave energy harvesting. Although the approach is developed within the deep‐water framework, the concept of the PB phase can be extended to other regimes, where the PB phase may exhibit complex dependence on the rotation angle of the scatterers, and the performance of the approach will be depth‐dependent. The PB phase properties and associated water wave manipulation under different depth conditions remains a valuable direction for future exploration. While the PB phase is inherently non‐dispersive due to its geometric nature, the proposed approach generally requires a working wavelength larger than two times the geometric size of the scatterers, with the upper bound determined by the desired deflection/focusing efficiency (a larger wavelength leads to a lower efficiency). The working bandwidth can be improved by optimizing the structural design of the scatterers, which is another interesting direction for future research. Akin to the optical PB phase,^[^ ^54^, ^55^, ^56^ ^]^ the new PB phase can be employed to investigate the geometric and topological properties of water waves.^[^ ^23^, ^24^ ^]^ Our work reveals the universality of the PB phase across different wave systems and in higher‐dimensional state evolutions, which may inspire the exploration of geometric phases in other high‐dimensional systems. The mechanism of the higher‐order unidirectional PB phase can be extended to other types of classical waves, such as surface plasmon polaritons and surface acoustic waves, offering a general and practical approach for controlling the phase of surface waves.

## 4 Calculation Methods

### Scattering Coefficients and Angular Momentum Selection Rule

The water waves are governed by the Laplace equation

(5) $$\nabla^{2}\Psi =0,$$

where Ψ(x,y,z) is the velocity potential. The above equation is subjected to the boundary conditions at y=0 (i.e., water‐air interface):^[^ ^34^ ^]^

(6) $$\frac{\partial }{\partial y}\Psi =\frac{\omega^{2}}{g}\Psi ,$$

and deep into water:

(7) $$\lim_{y\to -\infty }\frac{\partial }{\partial y}\Psi =0,$$

where *g* = 9.8 m · s^−2^ is the gravitational acceleration.^[^ ^34^ ^]^ The solution of the above equation is

(8) $$\Psi =e^{i\left(k_{x}x+k_{z}z\right)+ky},$$

where the propagation constant $k=\sqrt{k_{x}^{2}+k_{z}^{2}}$ satisfies the dispersion relation $\omega =\sqrt{\mathrm{gk}}$ under the deep‐water approximation. We neglect the time harmonic factor $e^{-i\omega t}$ throughout the paper. For the water wave propagating in ±x direction, the velocity potential is $\Psi =e^{\pm \mathrm{ikx}+\mathrm{ky}}$. The corresponding velocity field can be expressed as

(9) $$\left|v\right\rangle =\nabla \Psi \propto \left(\hat{x}+i\sigma \hat{y}\right)e^{-i\sigma kx+ky},$$

which is the eigenmode of the z‐component spin‐1 matrix operator:^[^ ^39^ ^]^ ${\hat{S}}_{z}\left|v\right\rangle =\sigma |v\rangle$, with eigenvalue σ=±1, i.e., the velocity field carries a spin in ±z direction. In the local polar coordinates system (r,α) centered at the scatterer, the velocity field can be expressed as

(10) $$\begin{array}{cc} \left|v\right\rangle & =\left(\hat{x}+i\sigma \hat{y}\right)e^{-i\sigma k\left(r\cos\alpha +x_{0}\right)+k\left(r\sin\alpha +y_{0}\right)}\\ & =I\left(\hat{x}+i\sigma \hat{y}\right)e^{-i\sigma kre^{i\sigma \alpha }}, \end{array}$$

where $I=e^{-i\sigma kx_{0}+ky_{0}}$, and $\left(x_{0},y_{0}\right)=\left(x_{0},-d\right)$ is the location of the scatterer's center in the global Cartesian coordinate system. For the scatterers that are small compared to the wavelength, the local velocity field |v⟩ around the scatterer can be expanded into partial waves as [40]:

(11) $$\left|v\right\rangle =\sum_{n=0}^{+\infty }\left|v_{n}\right\rangle =\sum_{n=0}^{+\infty }I\left(\hat{x}+i\sigma \hat{y}\right)\frac{{\left(-i\sigma kr\right)}^{n}e^{i\sigma n\alpha }}{n!},$$

where n is a non‐negative integer. The partial waves $|v_{n}\rangle$ represent vector vortex modes carrying both spin and OAM.

The scattering coefficients can be expressed as

(12) $$\tau^{f,b}=\langle v^{f,b}|\hat{T}\left|v^{i}\right\rangle ,$$

where $|v^{f}\rangle =\left(\hat{x}-i\hat{y}\right)e^{ikx+ky}$, $|v^{b}\rangle =\left(\hat{x}+i\hat{y}\right)e^{-ikx+ky}$ and $|v^{i}\rangle =\left(\hat{x}-i\hat{y}\right)e^{ikx+ky}$ are the forward scattering field, backward scattering field, and incident field respectively; $\hat{T}$ characterizes the interaction of the incident field $|v^{i}\rangle$ with the scatterer in the scatterer's local frame. Using Equation (11), we can write $|v^{i}\rangle =\sum_{n}|v_{n}^{i}\rangle ,|v^{f}\rangle =\sum_{n^{\prime }}\left|v_{n^{\prime }}^{f}\right\rangle$, and $|v^{b}\rangle =\sum_{n^{\prime }}\left|v_{n^{\prime }}^{b}\right\rangle$, thereby

(13) $$\tau^{f,b}=\sum_{n,n^{\prime }}\langle v_{n^{\prime }}^{f,b}|\hat{T}\left|v_{n}^{i}\right\rangle .$$

For a rotation of the scatterer by angle Δα, the scattering coefficients change to

(14) $$\tau^{f,b}\left(\Delta \alpha \right)=\sum_{n,n^{\prime }}\langle v_{n^{\prime }}^{f,b}|{\hat{R}}_{z}\left(\Delta \alpha \right)\hat{T}{\hat{R}}_{z}\left(-\Delta \alpha \right)\left|v_{n}^{i}\right\rangle .$$

Since the partial waves are the eigenmodes of the TAM operator ${\hat{J}}_{z}={\hat{S}}_{z}+{\hat{L}}_{z}$, application of the rotation operator ${\hat{R}}_{z}\left(-\Delta \alpha \right)$ results in the transformations: ${\hat{R}}_{z}\left(-\Delta \alpha \right)|v_{n}^{i}\rangle =e^{i\Delta \alpha j_{n}^{i}}\left|v_{n}^{i}\right\rangle$, ${\hat{R}}_{z}\left(-\Delta \alpha \right)|v_{n^{\prime }}^{f}\rangle =e^{i\Delta \alpha j_{n^{\prime }}^{f}}\left|v_{n^{\prime }}^{f}\right\rangle$ and ${\hat{R}}_{z}\left(-\Delta \alpha \right)|v_{n^{\prime }}^{b}\rangle =e^{i\Delta \alpha j_{n^{\prime }}^{b}}\left|v_{n^{\prime }}^{b}\right\rangle$, where $j_{n}^{i}=-1-n$, $j_{n^{\prime }}^{f}=-1-n^{\prime }$ and $j_{n^{\prime }}^{b}=1+n^{\prime }$. We note that $j_{n}^{i}$ and $j_{n^{\prime }}^{f}$ are negative, but $j_{n^{\prime }}^{b}$ is positive. The Hermitian conjugate versions of the transformations are: $\langle v_{n^{\prime }}^{f}|{\hat{R}}_{z}\left(\Delta \alpha \right)=$ 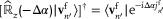 and $\langle v_{n^{\prime }}^{b}|{\hat{R}}_{z}\left(\Delta \alpha \right)=\left\langle v_{n^{\prime }}^{b}\right|e^{-i\Delta \alpha j_{n^{\prime }}^{b}}$. Substituting the above transformations into Equation (14), we can obtain

(15) $$\tau^{f,b}\left(\Delta \alpha \right)=\sum_{n,n^{\prime }}\langle v_{n^{\prime }}^{f,b}|\hat{T}\left|v_{n}^{i}\right\rangle e^{i\Delta \alpha (j_{n}^{i}-j_{n^{\prime }}^{f,b})}.$$

Since the scatterer with *C*_*m*_ symmetry is invariant under a rotation of 2π/m, we have $\tau^{f,b}\left(2\pi /m\right)=\tau^{f,b}\left(0\right)$. This indicates that

(16) $$j_{n^{\prime }}^{f,b}-j_{n}^{i}=Nm\;\left(N\in Z\right).$$

Equation (16) gives the angular momentum selection rule for the excitation of the partial waves. The integer *N* denotes the order of the angular momentum transfer channel (similar to the diffraction order in grating theory), which arises from the discrete rotational symmetry of the scatterer.

Equation (11) indicates that the partial wave amplitude $\left|\right|v_{n}\rangle |\propto {\left(\mathrm{kr}\right)}^{n}/n!$, where kr≪1, which implies that high‐order partial waves have very small amplitude in the region of the subwavelength scatterers. The scattering contributed by the channel $n\to n^{\prime }$ is $|\tau^{f,b}{|}_{n\to n^{\prime }}\propto \left||v_{n\prime }^{f,b}\rangle \right|\left||v_{n}^{i}\rangle \right|\propto {\left(kr\right)}^{n^{\prime }+n}/\left(n!n^{\prime }!\right)$. Consequently, the partial waves with the minimum $n+n^{\prime }$ that satisfy Equation (16) will dominate the scattering. For the forward scattering under *N* = 0 we have $n^{\prime }=n$ and thus $n^{\prime }+n=2n$. The dominant partial waves are $n^{\prime }=n=0$. For the forward scattering under |*N*| ⩾ 1, we have $n^{\prime }+n\geq \left|N\right|m$, which correspond to higher‐order partial waves that are negligible. For the backward scattering, we have $n^{\prime }+n=-n+\mathrm{Nm}-2+n=\mathrm{Nm}-2$ with *N* ⩾ 1. When *m* ⩾ 2, the dominating partial waves satisfy $n^{\prime }+n=m-2$ under *N* = 1; when *m* = 1, the dominating partial waves satisfy $n^{\prime }+n=2m-2=0$ under *N* = 2.

In summary, for the forward‐scattering partial waves, we have *N* = 0, and their TAM is

(17) $$j_{n^{\prime }}^{f}=j_{n}^{i}.$$

For backward‐scattering partial waves, we have *N* = 2 for *m* = 1 and *N* = 1 for *m* ⩾ 2. Thus, the TAM of the backward‐scattering partial waves is

(18) $$j_{n^{\prime }}^{b}=\left\{\begin{array}{cc} j_{n}^{i}+2, & m=1;\\ j_{n}^{i}+m, & m\geq 2. \end{array}\right.$$

Finally, substituting Equations (17) and (18) to Equation (15), we obtain the forward and backward scattering coefficients

(19) $$\begin{array}{c} \tau^{f}\left(\Delta \alpha \right)=\sum_{j_{n^{\prime }}^{f}-j_{n}^{i}=0}\langle v_{n^{\prime }}^{f}|\hat{T}\left|v_{n}^{i}\right\rangle , \end{array}$$

(20) $$\begin{array}{c} \tau^{b}\left(\Delta \alpha \right)=\left\{\begin{array}{cc} \langle v_{0}^{b}|\hat{T}\left|v_{0}^{i}\right\rangle e^{-i2\Delta \alpha }, & m=1;\\ \sum_{j_{n^{\prime }}^{b}-j_{n}^{i}=m}\langle v_{n^{\prime }}^{b}|\hat{T}\left|v_{n}^{i}\right\rangle e^{-im\Delta \alpha }, & m\geq 2. \end{array}\right. \end{array}$$

### Numerical Simulation

The full‐wave water wave simulations are performed with the package COMSOL Multiphysics using the Linearized Potential Flow module. The governing equation solved is the Laplace equation for the velocity potential, $\nabla^{2}\Psi =0$, under the assumptions of linear, inviscid, and irrotational wave. The equation is subject to the linearized free surface boundary condition $\frac{\partial }{\partial y}\Psi =\frac{\omega^{2}}{g}\Psi$ at the water‐air interface, along with impermeable boundary condition $\frac{\partial \Psi }{\partial n}=0$ at the surfaces of the scatterers. Radiation boundary condition is implemented at the computational domain boundaries to simulate an open environment. The wavelength is 62.5 mm in all the cases. The scatterers have prefect rigid boundary. For the simulations in Figure 1, we set *a* = 12.5 mm, *b* = 2.9 mm, *d* = 11 mm for the scatterer with *m* = 2, and *c* = 10.5 mm, *d* = 15 mm for the scatterers with *m* = 3 to 5. The depth *d* affects the amplitudes of the scattering coefficient but does not affect the geometric phase (Note S2, Supporting Information). For the simulations of the metasurfaces in Figure 4, the parameters *a*, *b* and *c* are the same as those in Figure 1. We set the depth *d* = 7.5 mm for the meta‐atoms with *m* = 2, and *d* = 11.5 mm for the meta‐atoms with *m* = 3 to 5. The periods of the metasurfaces in Figure 4b,d are *p* = 7.8 mm and *p* = 23.3 mm, respectively.

## 5 Experimental Section

The experiment is performed in a transparent tank (size 1 m × 1 m × 0.2 m) filled with water to a depth larger than half of a wavelength. We use sponges attached to the tank walls to mimic a reflectionless boundary for the water waves. The metasurface samples are fabricated by 3D‐printing technology using photosensitive resin. The parameters of the samples are *a* = 12.5 mm, *b* = 2.9 mm for the meta‐atoms with *m* = 2, and *c* = 10.5 mm for the meta‐atoms with *m* = 3 to 5. The metasurfaces in Figure 4b have the same rotation angle profile with Δ*α*(*z* − *p*) − Δ*α*(*z*) = 6° and the period *p* = 7.8 mm. The metasurfaces in Figure 4d have different rotation angle profiles with the same period *p* = 23.3 mm. A ripple generator is used to generate the plane wave with desired frequency. The amplitude of the generated surface elevation is smaller than the shortest distance from the sample to the mean surface elevation of the water. We use a light source locating above the tank to project the water wave pattern onto a plane beneath, which can be directly observed and video‐recorded for analysis. To record the pattern of the reflected waves only, we stop the ripple generator after running it for several periods. A high‐resolution video camera (OmniVision OV64B, 64 MP) is used to capture the surface wave propagation. The stationary background is subtracted from the video by using the Fast Principal Component Pursuit algorithm implemented in the open‐source LRSLibrary toolbox.^[^ ^57^ ^]^

## Conflict of Interest

The authors declare no conflict of interest.

## Author Contributions

S.W. conceived the idea and supervised the project. W.X. conducted the numerical simulations and performed the experiments. S.J. and T.F. assisted in the experiments. W.X. and S.W. wrote the draft. All authors contributed to discussions, interpretation of the results, and polishing of the manuscript.

## Supporting information

Supporting Information

## Data Availability

The data that support the findings of this study are available from the corresponding author upon reasonable request.
