# Supplementary material for: Water‐Wave Pancharatnam‐Berry Phase Induced by 4D Spin‐Orbit State Evolution
Source: Adv Sci (Weinh). 2025 Oct 13;13(2):e15337. doi: 10.1002/advs.202515337 (PMC12786372; doi:10.1002/advs.202515337)
Supplement: Supplementary file 1 — Supporting Information [file ADVS-13-e15337-s001.pdf]

# Supplementary Information for
# Water-wave Pancharatnam-Berry phase induced by four-dimensional spin-orbit state evolution

Wanyue Xiao,[1] Shiqi Jia,[1] Tong Fu,[1] and Shubo Wang[1,*]

[1] *Department of Physics, City University of Hong Kong, Tat Chee Avenue, Kowloon, Hong Kong, China*

**Contents**

**NOTE 1. Partial wave analysis for the backward scattering waves** 2

**NOTE 2. Scattering coefficients for scatterers at different depths** 2

**NOTE 3. Partial wave analysis for the velocity fields of the scatterers** 3

**NOTE 4. Numerical calculation of the couplings from multipoles to water wave** 4

**References** 5

## NOTE 1. PARTIAL WAVE ANALYSIS FOR THE BACKWARD SCATTERING WAVES

Equation (18) indicates that the lowest-order scattering channel for the $C_1$ (or $C_2$) scatterers is $j_0^{\mathrm{i}} = -1\,(l=0,\sigma=-1) \to j_0^{\mathrm{b}} = 1\,(l=0,\sigma=1)$. The corresponding scattering coefficient is $\tau^{\mathrm{b}} = \langle \mathbf{v}_0^{\mathrm{b}}|\hat{T}|\mathbf{v}_0^{\mathrm{i}}\rangle \mathrm{e}^{-i2\Delta\alpha}$ with the PB phase $\varphi_{\mathrm{PB}} = -2\Delta\alpha = (j_0^{\mathrm{i}} - j_0^{\mathrm{b}})\Delta\alpha = (\sigma^{\mathrm{i}} - \sigma^{\mathrm{b}})\Delta\alpha$. Thus, the PB phase induced by $C_1$ (or $C_2$ ) scatterers is solely decided by the differential spin of $|\mathbf{v}_0^{\mathrm{i}}\rangle$ and $|\mathbf{v}_0^{\mathrm{b}}\rangle$.

For the scatterers with $C_3$ symmetry, there are two lowest-order scattering channels: $j_0^{\mathrm{i}} = -1\ (l=0,\sigma=-1) \to j_1^{\mathrm{b}} = 2\,(l=1,\sigma=1)$ and $j_1^{\mathrm{i}} = -2\,(l=-1,\sigma=-1) \to j_0^{\mathrm{b}} = 1\,(l=0,\sigma=1)$. The corresponding scattering coefficient is $\tau^{\mathrm{b}} = \langle \mathbf{v}_1^{\mathrm{b}}|\hat{T}|\mathbf{v}_0^{\mathrm{i}}\rangle \mathrm{e}^{-i3\Delta\alpha} + \langle \mathbf{v}_0^{\mathrm{b}}|\hat{T}|\mathbf{v}_1^{\mathrm{i}}\rangle \mathrm{e}^{-i3\Delta\alpha}$ with the PB phase $\varphi_{\mathrm{PB}} = (j_0^{\mathrm{i}} - j_1^{\mathrm{b}})\Delta\alpha = (j_1^{\mathrm{i}} - j_0^{\mathrm{b}})\Delta\alpha = -3\Delta\alpha$. In this case, the PB phase is contributed by both spin and OAM.

For the scatterer with $C_4$ symmetry, there are three lowest-order scattering channels: $j_0^{\mathrm{i}} = -1\,(l=0,\sigma=-1) \to j_2^{\mathrm{b}} = 3\,(l=2,\sigma=1)$, $j_1^{\mathrm{i}} = -2\,(l=-1,\sigma=-1) \to j_1^{\mathrm{b}} = 2\,(l=1,\sigma=1)$ and $j_2^{\mathrm{i}} = -3\,(l=-2,\sigma=-1) \to j_0^{\mathrm{b}} = 1\,(l=0,\sigma=1)$. The corresponding scattering coefficient is $\tau^{\mathrm{b}} = \langle \mathbf{v}_2^{\mathrm{b}}|\hat{T}|\mathbf{v}_0^{\mathrm{i}}\rangle \mathrm{e}^{-i4\Delta\alpha} + \langle \mathbf{v}_1^{\mathrm{b}}|\hat{T}|\mathbf{v}_1^{\mathrm{i}}\rangle \mathrm{e}^{-i4\Delta\alpha} + \langle \mathbf{v}_0^{\mathrm{b}}|\hat{T}|\mathbf{v}_2^{\mathrm{i}}\rangle \mathrm{e}^{-i4\Delta\alpha}$ with the PB phase $\varphi_{\mathrm{PB}} = (j_0^{\mathrm{i}} - j_2^{\mathrm{b}})\Delta\alpha = (j_1^{\mathrm{i}} - j_1^{\mathrm{b}})\Delta\alpha = (j_2^{\mathrm{i}} - j_0^{\mathrm{b}})\Delta\alpha = -4\Delta\alpha$.

For the scatterer with $C_5$ symmetry, there are four lowest-order scattering channels: $j_0^{\mathrm{i}} = -1\,(l=0,\sigma=-1) \to j_3^{\mathrm{b}} = 4\,(l=3,\sigma=1)$, $j_1^{\mathrm{i}} = -2\,(l=-1,\sigma=-1) \to j_2^{\mathrm{b}} = 3\,(l=2,\sigma=1)$, $j_2^{\mathrm{i}} = -3\,(l=-2,\sigma=-1) \to j_1^{\mathrm{b}} = 2\,(l=1,\sigma=1)$ and $j_3^{\mathrm{i}} = -4\,(l=-3,\sigma=-1) \to j_0^{\mathrm{b}} = 1(l=0,\sigma=1)$. The corresponding scattering coefficient is $\tau^{\mathrm{b}} = \langle \mathbf{v}_3^{\mathrm{b}}|\hat{T}|\mathbf{v}_0^{\mathrm{i}}\rangle \mathrm{e}^{-i5\Delta\alpha} + \langle \mathbf{v}_2^{\mathrm{b}}|\hat{T}|\mathbf{v}_1^{\mathrm{i}}\rangle \mathrm{e}^{-i5\Delta\alpha} + \langle \mathbf{v}_1^{\mathrm{b}}|\hat{T}|\mathbf{v}_2^{\mathrm{i}}\rangle \mathrm{e}^{-i5\Delta\alpha} + \langle \mathbf{v}_0^{\mathrm{b}}|\hat{T}|\mathbf{v}_3^{\mathrm{i}}\rangle \mathrm{e}^{-i5\Delta\alpha}$ with the PB phase $\varphi_{\mathrm{PB}} = (j_0^{\mathrm{i}} - j_3^{\mathrm{b}})\Delta\alpha = (j_1^{\mathrm{i}} - j_2^{\mathrm{b}})\Delta\alpha = (j_2^{\mathrm{i}} - j_1^{\mathrm{b}})\Delta\alpha = (j_3^{\mathrm{i}} - j_0^{\mathrm{b}})\Delta\alpha = -5\Delta\alpha$.

The above analysis is shown in Table. S1. As seen, for the scatterers with higher rotational symmetries, the partial waves with larger OAM differences will be involved in the scattering process, leading to a larger PB phase under the same rotation angle $\Delta\alpha$.

TABLE. S1: **Partial wave analysis.**

| $C_m$ | $\lvert\mathbf{v}_n^{\mathrm{i}}\rangle$ | $l$ | $\sigma$ | $j_n^{\mathrm{i}}$ | $\lvert\mathbf{v}_{n'}^{\mathrm{b}}\rangle$ | $l$ | $\sigma$ | $j_{n'}^{\mathrm{b}}$ | $\varphi_{\mathrm{PB}}$ |
|---|---|---|---|---|---|---|---|---|---|
| $m=1,2$ | $\lvert\mathbf{v}_0^{\mathrm{i}}\rangle$ | 0 | -1 | -1 | $\lvert\mathbf{v}_0^{\mathrm{b}}\rangle$ | 0 | 1 | 1 | $-2\Delta\alpha$ |
| $m=3$ | $\lvert\mathbf{v}_0^{\mathrm{i}}\rangle$ | 0 | -1 | -1 | $\lvert\mathbf{v}_1^{\mathrm{b}}\rangle$ | 1 | 1 | 2 | $-3\Delta\alpha$ |
| | $\lvert\mathbf{v}_1^{\mathrm{i}}\rangle$ | -1 | -1 | -2 | $\lvert\mathbf{v}_0^{\mathrm{b}}\rangle$ | 0 | 1 | 1 | |
| $m=4$ | $\lvert\mathbf{v}_0^{\mathrm{i}}\rangle$ | 0 | -1 | -1 | $\lvert\mathbf{v}_2^{\mathrm{b}}\rangle$ | 2 | 1 | 3 | $-4\Delta\alpha$ |
| | $\lvert\mathbf{v}_1^{\mathrm{i}}\rangle$ | -1 | -1 | -2 | $\lvert\mathbf{v}_1^{\mathrm{b}}\rangle$ | 1 | 1 | 2 | |
| | $\lvert\mathbf{v}_2^{\mathrm{i}}\rangle$ | -2 | -1 | -3 | $\lvert\mathbf{v}_0^{\mathrm{b}}\rangle$ | 0 | 1 | 1 | |
| $m=5$ | $\lvert\mathbf{v}_0^{\mathrm{i}}\rangle$ | 0 | -1 | -1 | $\lvert\mathbf{v}_3^{\mathrm{b}}\rangle$ | 3 | 1 | 4 | $-5\Delta\alpha$ |
| | $\lvert\mathbf{v}_1^{\mathrm{i}}\rangle$ | -1 | -1 | -2 | $\lvert\mathbf{v}_2^{\mathrm{b}}\rangle$ | 2 | 1 | 3 | |
| | $\lvert\mathbf{v}_2^{\mathrm{i}}\rangle$ | -2 | -1 | -3 | $\lvert\mathbf{v}_1^{\mathrm{b}}\rangle$ | 1 | 1 | 2 | |
| | $\lvert\mathbf{v}_3^{\mathrm{i}}\rangle$ | -3 | -1 | -4 | $\lvert\mathbf{v}_0^{\mathrm{b}}\rangle$ | 0 | 1 | 1 | |

## NOTE 2. SCATTERING COEFFICIENTS FOR SCATTERERS AT DIFFERENT DEPTHS

In Fig. S1a to d, we show the numerically simulated scattering coefficients for the scatterers with different rotational symmetries $C_m$ at different depths $d_1 < d_2 < d_3$. As seen, the amplitudes of the scattering coefficients decrease as the depth increases. This is because that the water waves are confined at the water-air interface, thus, the wave-scatterer interaction is weaker when the scatterer locates far from the interface. When the scatterer is close to the water-air interface, the reflection of the scatterer's fields from the interface is strong, which can affect the excitation of the scatterer (a back-reaction effect). Therefore, the scattering amplitudes vary with the rotation angles and exhibit non-uniformity. Specially, the scattering amplitudes reach their maxima at the rotation angle that gives the shortest distance between the scatterer and the mean surface elevation of the water. When the scatterer is submerged at a large depth, the effect of the reflective evanescent fields is suppressed, and the amplitudes of the scattering coefficients become uniform, as predicted by Eqs. (19) and (20). On the other hand, the relationship between the phases of the scattering coefficients and the rotation angle is insensitive to the depth of the scatterer, as shown in Fig. S1.

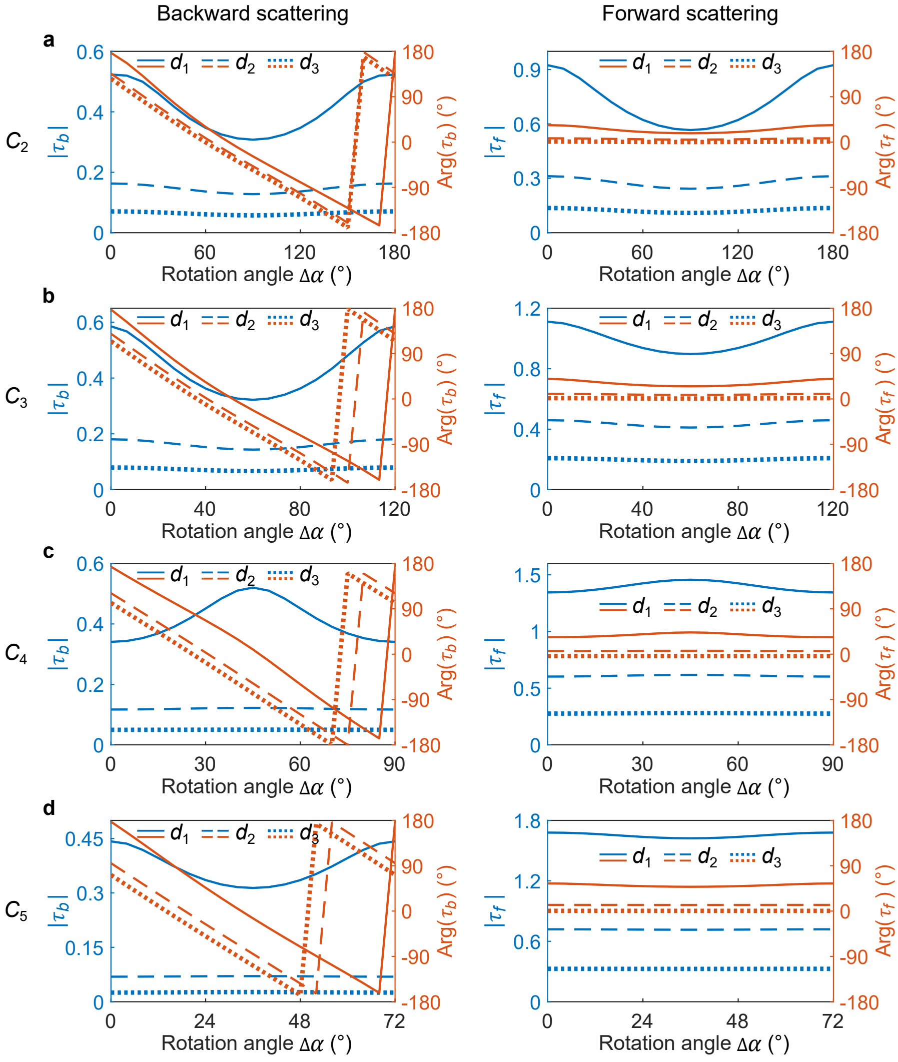


FIG. S1: Scattering coefficients for the scatterers at different depths. For $m = 2$: $d_1 = 7$ mm, $d_2 = 11$ mm, $d_3 = 15$ mm; for $m = 3$ to 5: $d_1 = 11$ mm, $d_2 = 15$ mm, $d_3 = 19$ mm.

## NOTE 3. PARTIAL WAVE ANALYSIS FOR THE VELOCITY FIELDS OF THE SCATTERERS

Under the excitation of the incident water wave, the scatterer will generate scattering field, which can be determined by using the boundary element method [1]:

$$\Psi(\mathbf{r}) = \int_{\partial^2\tau} \Psi_t\left(\mathbf{r}'\right)\mathbf{n}'\cdot\nabla' G\left(\mathbf{r},\mathbf{r}'\right) - \mathbf{n}'\cdot\nabla'\Psi_t\left(\mathbf{r}'\right)G\left(\mathbf{r},\mathbf{r}'\right)dr', \tag{S1}$$

where $\mathbf{n}'$ is the unit normal vector on the boundary of the scatterer (denoted by $\partial^2\tau$), and $\Psi_t\left(\mathbf{r}'\right)$ is the total velocity potential at the boundary. We note that $\Psi_t\left(\mathbf{r}'\right)\mathbf{n}'$ and $\mathbf{n}'\cdot\nabla'\Psi_t\left(\mathbf{r}'\right)$ correspond to the 'dipole' (vector) and 'monopole' (scalar) boundary source densities, respectively. Since the scatterers have prefect rigid boundary (Neumann-type boundary), the normal component of the velocity field at the boundary is zero, i.e., $v_\perp \propto \mathbf{n}'\cdot\nabla'\Psi_t = 0$, and only the dipole source exists at the boundary.

In Eq. (S1), $G\left(\mathbf{r},\mathbf{r}'\right) = -\ln\left(|\mathbf{r}-\mathbf{r}'|\right)/(2\pi)$ is the 2D free-space Green's function [2], which satisfies the Poisson equation $\nabla^2 G\left(\mathbf{r},\mathbf{r}'\right) = -\delta\left(\mathbf{r},\mathbf{r}'\right)$ under the radiation boundary condition. The Green function can be expanded as [2]

$$G\left(\mathbf{r},\mathbf{r}'\right) = \frac{-\ln r}{2\pi} + \frac{1}{2\pi}\sum_{n=1}^{+\infty}\frac{1}{n}\left(\frac{r'}{r}\right)^n \cos\left[n\left(\alpha-\alpha'\right)\right]. \tag{S2}$$

Substituting Eq. (S2) into Eq. (S1) and only consider the contribution of the dipole source $\Psi_t(\mathbf{r}')\,\mathbf{n}'$, we can obtain the partial wave expansions (i.e., multipole expansions) of the scatterer's velocity potential

$$\Psi(\mathbf{r}) = \sum_{n=1}^{+\infty} \frac{-1}{2nk^n r^n}\left(a_n \mathrm{e}^{in\alpha} + b_n \mathrm{e}^{-in\alpha}\right), \tag{S3}$$

where

$$a_n = \frac{1}{4\pi}\int_{\partial^2\tau} -2nk^n r'^{n-1} \mathrm{e}^{-i(n-1)\alpha'} [\Psi_t(\mathbf{r}')\, n_{x'} - i\Psi_t(\mathbf{r}')\, n_{y'}] dr', \tag{S4}$$

and

$$b_n = \frac{1}{4\pi}\int_{\partial^2\tau} -2nk^n r'^{n-1} \mathrm{e}^{i(n-1)\alpha'} [\Psi_t(\mathbf{r}')\, n_{x'} + i\Psi_t(\mathbf{r}')\, n_{y'}] dr' \tag{S5}$$

are the complex amplitudes of the multipoles. The corresponding velocity field is

$$|\gamma\rangle = \nabla\Psi = \sum_{n=1}^{+\infty} \frac{1}{2k^n r^{n+1}} \left\{ \begin{array}{c} \left[a_n \mathrm{e}^{i(n+1)\alpha} + b_n \mathrm{e}^{-i(n+1)\alpha}\right]\hat{\mathbf{x}} \\ + \left[-ia_n \mathrm{e}^{i(n+1)\alpha} + ib_n \mathrm{e}^{-i(n+1)\alpha}\right]\hat{\mathbf{y}} \end{array} \right\}. \tag{S6}$$

Adopting the circular polarization basis, the above velocity field can be rewritten as:

$$|\gamma\rangle = \sum_{n=1}^{+\infty} a_n(\hat{\mathbf{x}} - i\hat{\mathbf{y}})\frac{\mathrm{e}^{i(n+1)\alpha}}{k^n r^{n+1}} + b_n(\hat{\mathbf{x}} + i\hat{\mathbf{y}})\frac{\mathrm{e}^{-i(n+1)\alpha}}{k^n r^{n+1}}. \tag{S7}$$

## NOTE 4. NUMERICAL CALCULATION OF THE COUPLINGS FROM MULTIPOLES TO WATER WAVE

The fields of the scatterer can be decomposed into multipoles as detailed in last section. The couplings of these multipoles to the water waves can be numerically calculated by simulating the fields excited by the multipoles. The multipoles are extracted with Eqs. (S4)-(S7) and then constructed in COMSOL by arranging $Q$ monopole point sources on a ring of radius $R$. The distribution of the monopole point sources can be expressed as

$$\lambda_n(\mathbf{r}') = \frac{2\pi}{Qk^n r'^n} a_n e^{in\alpha'} \delta(r', R)\, \delta\left(\alpha', q\frac{2\pi}{Q}\right), \tag{S8}$$

where $\delta(r', R)$ and $\delta\left(\alpha', q\frac{2\pi}{Q}\right)$ are the Kronecker delta with $q = 1, 2, \ldots, Q$ denoting the $q$th monopole source. The resulting field $\Psi_n(\mathbf{r})$ can be obtained as [2]:

$$\Psi_n(\mathbf{r}) = -\int_{\partial\tau} \lambda_n(\mathbf{r}')\, G(\mathbf{r}, \mathbf{r}')\, dr', \tag{S9}$$

where the Green's function $G(\mathbf{r}, \mathbf{r}')$ is given in Eq. (S2). Substituting Eq. (S8) into Eq. (S9), we can obtain

$$\begin{gathered} \Psi_n(\mathbf{r}) = \frac{-a_n \ln r}{Qk^n R} \sum_{q=1}^{Q} \mathrm{e}^{inq\frac{2\pi}{Q}} + \\ \frac{-a_n}{Qk^n} \sum_{s=1}^{\infty} \int_{\partial\tau} \mathrm{e}^{in\alpha'} \left[ \begin{array}{c} \frac{\cos(s\alpha)}{2sr^s}(\mathrm{e}^{is\alpha'} + \mathrm{e}^{-is\alpha'}) \\ + \frac{\sin(s\alpha)}{2isr^s}(\mathrm{e}^{is\alpha'} - \mathrm{e}^{-is\alpha'}) \end{array} \right] \delta(r', R)\, \delta\left(\alpha', q\frac{2\pi}{Q}\right) dr'. \end{gathered} \tag{S10}$$

Using

$$\int_{\partial\tau} \mathrm{e}^{-is\alpha'} \mathrm{e}^{in\alpha'} \delta(r', R)\, \delta\left(\alpha', q\frac{2\pi}{Q}\right) dr' = \sum_{q=1}^{Q} \mathrm{e}^{i(n-s)q\frac{2\pi}{Q}} = Q\delta_{ns}, \tag{S11}$$

Eq. (S10) can be reduced to

$$\Psi_n(\mathbf{r}) = \frac{-a_n}{k^n}\frac{1}{2nr^n}[\cos(n\alpha) + i\sin(n\alpha)] = \frac{-a_n}{2nk^n r^n}\mathrm{e}^{in\alpha}. \tag{S12}$$

The corresponding velocity field generated by the point sources is $\nabla\Psi_n(\mathbf{r}) = a_n(\hat{\mathbf{x}} - i\hat{\mathbf{y}})\frac{\mathrm{e}^{i(n+1)\alpha}}{k^n r^{n+1}}$, which is equivalent to $|\gamma_n^+\rangle$. Thereby, any multipoles $|\gamma_n^+\rangle$ can be constructed with the monopole point sources $\lambda_n(\mathbf{r}')$. Similarly, the multipoles $|\gamma_n^-\rangle$ can also be constructed with the following monopole point sources:

$$\lambda_n(\mathbf{r}') = \frac{2\pi}{Qk^n r'^n} b_n \mathrm{e}^{-in\alpha'}\delta(r', R)\,\delta\left(\alpha', q\frac{2\pi}{Q}\right). \tag{S13}$$

The multipoles are constructed in COMSOL using the above ring of monopole point sources, which are positioned beneath the water-air interface, with its center coinciding with the center of the scatterer. The radius of the ring $R$ =1.3 mm is much smaller than the wavelength and the dimensions of the scatterers. We set $Q$ =72 for the considered scatterers to ensure accuracy. We then simulate the water surface wave field excited by the multipoles. The coupling coefficients are obtained by measuring the resulting velocity potential at the water-air interface (normalized by the amplitude of the incident wave).

* Electronic address: shubwang@cityu.edu.hk

[1] L. C. Wrobel, *The Boundary Element Method, Volume 1: Applications in Thermo-fluids and Acoustics* (John Wiley & Sons, 2002).
[2] J. D. Jackson, *Classical Electrodynamics* (John Wiley & Sons, 2021).
